# Supplementary material for: Glycated Albumin and Continuous Glucose Monitoring Metrics in Dogs with Diabetes Mellitus: A Pilot Study
Source: Animals (Basel). 2025 Jul 8;15(14):2004. doi: 10.3390/ani15142004 (PMC12291857; doi:10.3390/ani15142004)
Supplement: Supplementary file 1 [file animals-15-02004-s001.zip › animals-3653395-supplementary.pdf]

**Table S1. Evaluation of diabetes control**

| | | |
|---|---|---|
| Patient history (All refer to changes over previous 2 weeks) | | |
| 1 | Need for insulin adjustment | Yes<br>No |
| 2 | Change in water consumption | Yes-increased water consumption<br>No |
| 3 | Change in urine frequency/amount | Yes<br>No |
| 4 | Change in appetite/food intake | Change in appetite (increased)<br>No change in appetite |
| 5 | Change in activity level | Change in activity level (decreased)<br>No change in activity level |
| 6 | Increase in body weight | Yes<br>No (no change/decrease) |
| 7 | Signs of hypoglycemia noticed | Yes<br>No |
| 8 | Required treatment for hypoglycemia | Yes<br>No |
| 9 | Compliant with recommended insulin dosing | Yes<br>No |
| Physical examination | | |
| 10 | Change body weight ≥ 5% | Yes<br>No |
| 11 | Body condition score | Unacceptable (poor body condition)<br>Acceptable (good body condition) |
| 12 | Hydration status | Dehydrated<br>Normal hydration |

**Table S2.** Signalment of dogs with diabetes mellitus and non-diabetic dogs. Breed, sex, and age of diabetic and control group dogs participating in this study

| **Diabetes mellitus group (n=10)** | | | | | | | |
|---|---|---|---|---|---|---|---|
| **No.** | **Breed** | **Sex** | **Age** | **No.** | **Breed** | **Sex** | **Age** |
| **1** | Maltese | MC | 13y | **6** | Korean Jindo | SF | 14y |
| **2** | Mixed | MC | 11y | **7** | Maltese | SF | 4y |
| **3** | Bichon Frise | F | 9y | **8** | Bichon Frise | F | 9y |
| **4** | Maltese | MC | 12y | **9** | Pomeranian | SF | 11y |
| **5** | Golden Retriever | SF | 9y | **10** | Poodle | SF | 14y |
| **Control group (n=20)** | | | | | | | |
| **No.** | **Breed** | **Sex** | **Age** | **No.** | **Breed** | **Sex** | **Age** |
| **1** | Bichon Frise | MC | 2y | **11** | Maltese | SF | 9y |
| **2** | Chihuahua | MC | 11y | **12** | Maltese | SF | 13y |
| **3** | Pomeranian | MC | 9y | **13** | Maltese | SF | 12y |
| **4** | Poodle | MC | 9y | **14** | Mixed | SF | 1y |
| **5** | Poodle | MC | 12y | **15** | Poodle | SF | 13y |
| **6** | Poodle | MC | 9y | **16** | Poodle | SF | 14y |
| **7** | Mixed | F | 1y | **17** | Poodle | SF | 12y |
| **8** | Pomeranian | F | 9y | **18** | Miniature Schnauzer | SF | 9y |
| **9** | French Bulldog | MC | 6y | **19** | Shih Tzu | SF | 7y |
| **10** | Dachshund | SF | 7y | **20** | Japanese Spitz | uk | 7y |

Abbreviations: CM, castrated male; F, female; M, male; SF, spayed female; uk, unknown.
